# Supplementary material for: Causal Relationship between Aging and Anorexia Nervosa: A White-Matter-Microstructure-Mediated Mendelian Randomization Analysis
Source: Biomedicines. 2024 Aug 16;12(8):1874. doi: 10.3390/biomedicines12081874 (PMC11351342; doi:10.3390/biomedicines12081874)
Supplement: Supplementary file 1 [file biomedicines-12-01874-s001.zip › Supplementary Figure/Figure S4/Singe SNP plot of MR analysis for TL to ubm-b-1659.pdf]

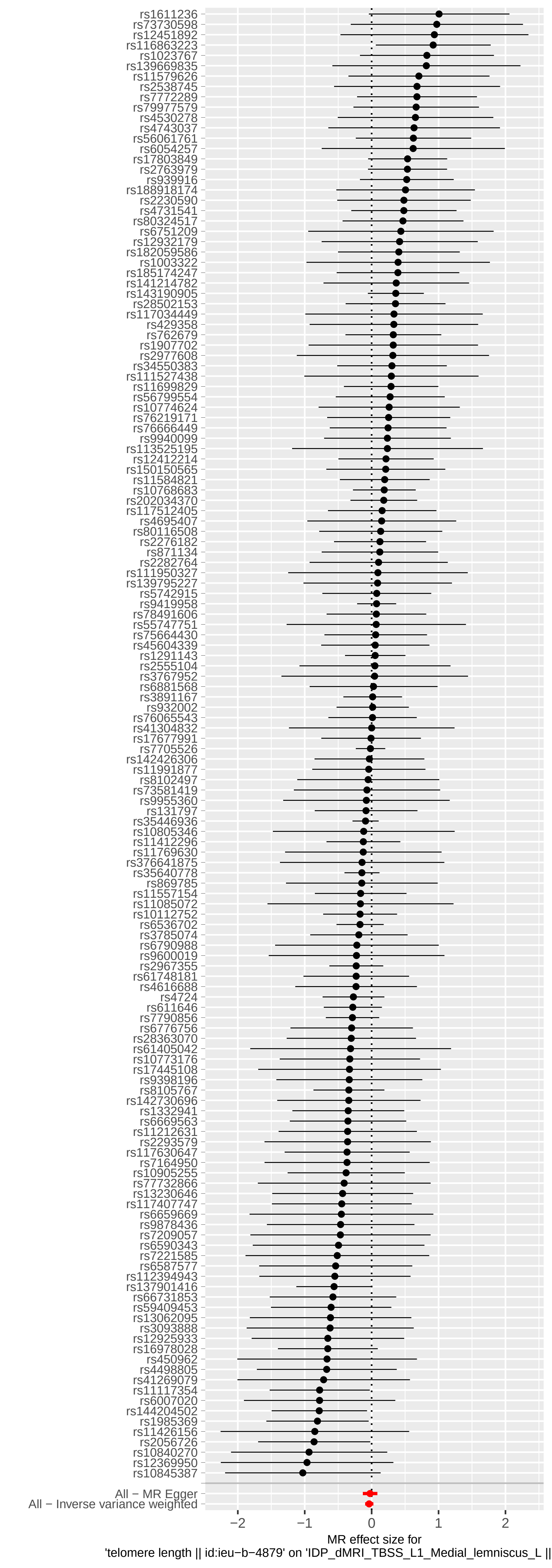

rs1611236
rs73730598
rs12451892
rs116863223
rs1023767
rs139669835
rs11579626
rs2538745
rs7772289
rs79977579
rs4530278
rs4743037
rs56061761
rs6054257
rs17803849
rs2763979
rs939916
rs188918174
rs2230590
rs4731541
rs80324517
rs6751209
rs12932179
rs182059586
rs1003322
rs185174247
rs141214782
rs143190905
rs28502153
rs117034449
rs429358
rs762679
rs1907702
rs2977608
rs34550383
rs111527438
rs11699829
rs56799554
rs10774624
rs76219171
rs76666449
rs9940099
rs113525195
rs12412214
rs150150565
rs11584821
rs10768683
rs202034370
rs117512405
rs4695407
rs80116508
rs2276182
rs871134
rs2282764
rs111950327
rs139795227
rs5742915
rs9419958
rs78491606
rs55747751
rs75664430
rs45604339
rs1291143
rs2555104
rs3767952
rs6881568
rs3891167
rs932002
rs76065543
rs41304832
rs17677991
rs7705526
rs142426306
rs11991877
rs8102497
rs73581419
rs9955360
rs131797
rs35446936
rs10805346
rs11412296
rs11769630
rs376641875
rs35640778
rs869785
rs11557154
rs11085072
rs10112752
rs6536702
rs3785074
rs6790988
rs9600019
rs2967355
rs61748181
rs4616688
rs4724
rs611646
rs7790856
rs6776756
rs28363070
rs61405042
rs10773176
rs17445108
rs9398196
rs8105767
rs142730696
rs1332941
rs6669563
rs11212631
rs2293579
rs117630647
rs7164950
rs10905255
rs77732866
rs13230646
rs117407747
rs6659669
rs9878436
rs7209057
rs6590343
rs7221585
rs6587577
rs112394943
rs137901416
rs66731853
rs59409453
rs13062095
rs3093888
rs12925933
rs16978028
rs450962
rs4498805
rs41269079
rs11117354
rs6007020
rs144204502
rs1985369
rs11426156
rs2056726
rs10840270
rs12369950
rs10845387
All − MR Egger
All − Inverse variance weighted
−2
−1
0
1
2
MR effect size for
'telomere length || id:ieu−b−4879' on 'IDP_dMRI_TBSS_L1_Medial_lemniscus_L ||
